# Norms and T-scores for screeners of alcohol use, depression and anxiety in the population of Suriname

**DOI:** 10.3389/fpsyt.2023.1088696

**Published:** 2023-04-27

**Authors:** Edwin de Beurs, Raj Jadnanansing, Kajal Etwaroo, Matthijs Blankers, Robbert Bipat, Jaap Peen, Jack Dekker

**Affiliations:** ^1^Department of Clinical Psychology, Leiden Universiteit, Leiden, Netherlands; ^2^Research department, Arkin Mental Health Care, Amsterdam, Netherlands; ^3^Department of Psysiology, Anton de Kom University, Tammenga, Suriname; ^4^Department of Psychiatry, Amsterdam University Medical Center, Amsterdam, Netherlands; ^5^Trimbos Institute, Utrecht, Netherlands; ^6^Department of Clinical Psychology, Vrije Universiteit, Amsterdam, Netherlands

**Keywords:** screening, alcohol use disorder, depression, anxiety, T-scores, norms

## Abstract

**Background:**

There is a considerable gap between care provision and the demand for care for common mental disorders in low-and-middle-income countries. Screening for these disorders, e.g., in primary care, will help to close this gap. However, appropriate norms and threshold values for screeners of common mental disorders are lacking.

**Methods:**

In a survey study, we gathered data on frequently used screeners for alcohol use disorders, (AUDIT), depression, (CES-D), and anxiety disorders (GAD-7, ACQ, and BSQ) in a representative sample from Suriname, a non-Latin American Caribbean country. A stratified sampling method was used by random selection of 2,863 respondents from 5 rural and 12 urban resorts. We established descriptive statistics of all scale scores and investigated unidimensionality. Furthermore, we compared scores by gender, age-group, and education level with *t*-test and Mann–Whitney U tests, using a significance level of *p* < 0.05.

**Results:**

Norms and crosswalk tables were established for the conversion of raw scores into a common metric: T-scores. Furthermore, recommended cut-off values on the T-score metric for severity levels were compared with international cut-off values for raw scores on these screeners.

**Discussion:**

The appropriateness of these cut-offs and the value of converting raw scores into T-scores are discussed. Cut-off values help with screening and early detection of those who are likely to have a common mental health disorder and may require treatment. Conversion of raw scores to a common metric in this study facilitates the interpretation of questionnaire results for clinicians and can improve health care provision through measurement-based care.

## Background

Common Mental Disorders (CMD’s), such as depression, anxiety, and Alcohol Use Disorders (AUD) are highly prevalent worldwide (1). Global year prevalence rates are about 4.7% for depressive disorders (2), about 7.3% for anxiety disorders (3) and about 5.0% for AUDs (4). These common mental disorders are significantly associated with impairment of quality of life, lower social functioning, and high societal costs (5). The 12-month prevalence of estimates of major depression, anxiety and AUDs are about the same in high-income as in Low-to-Middle-Income Countries (LMICs) (3, 6).

In recent decades, empirically supported psychological treatments have been developed for these CMDs with are highly efficacious and efficient (7). In a meta-analysis, these empirically psychological treatments for depression and anxiety were also effective in LMICs (8). Therefore, global dissemination of these interventions in LMICs is advocated by the WHO. However, in LMICs, the availability of these treatments is limited. Chisholm et al. (9) estimated intervention coverage in LMICs as 14 and 10% for depression and anxiety respectively, corresponding to a treatment gap of 86 to 90% for these disorders. According the World Health Organization, the treatment gap for mental disorders is 30–50% in developed countries and 76–80% in LMICs (10).

A major obstacle to widespread use of these short screeners is that norms and cut-off values for potential “caseness” for these screeners for the population of Suriname have not been determined. An additional problem is that each instrument has its own scale, norm scores and cut-off value. Direct comparisons of scores on dimensions such as depression, anxiety, and alcohol dependence are not easily done. In addition to calculating the norm values for the Surinamese population, we have also established crosswalks (conversion tables and a figure) to a common metric for these instruments, the T-score, applicable in this target group.

The traditional way to provide normative values for measurement instruments is to compose for each measure a set of tables for various groups of respondents (distinct by clinical status, gender, or age) with raw score ranges and their meaning in levels from very low to very high (see Table 1 in the present paper). In addition, data on clinically meaningful cut-off scores are provided, such as a cut-off score for clinical level or “caseness” and a cut-off score for reliable change, aka the Reliable Change Index (11). However, more and more we see an international trend towards scoring measures on a common metric, usually a standardized score. This allows researchers to gather and compare data from various studies more efficiently. For clinicians, such a common metric is convenient to interpret scores from various outcome scales more easily and relay information from test scores to their patients. The T-score has been chosen by the PROMIS group as the common metric and several papers have been published with cross-walk tables for frequently used measures of depression (12), anxiety (13), and psychological distress (14).

**Table 1:** Norms for all respondents, males, and females.

	AUDIT			CESD	GAD	ACQ			BSQ
Scale	TOT	USE	PRO			TOT	PHY	LC	
All									
Very low	0.00–0.99	0.00–0.99	0.00–3.99	0.00–0.99	0.00–0.99	1.00–1.06	1.00–1.13	1.00–1.28	1.00–1.05
Low									
Below average				1.00–3.99					
Average				4.00–7.99	1.00–2.99				1.06–1.36
Above average	1.00–3.99	1.00–2.99		8.00–13.99	3.00–5.99	1.07–1.28			1.37–2.05
High	4.00–8.99	3.00–5.99		14.00–28.99	6.00–12.99	1.29–1.83	1.14–1.85	1.29–1.99	2.06–3.34
Very high	9.00–40.00	6.00–12.00	4.00–28.00	29.00–80.00	13.00–21.00	1.84–5.00	1.86–5.00	2.00–5.00	3.35–5.00
Men									
Very low	0.00–1.99	0.00–1.99	1.00–1.99	0.00–0.99	0.00–0.99	1.00–1.14	1.00–1.13	1.00–1.13	1.00–1.28
Low									
Below average				1.00–3.99					
Average	2.00–3.99	1.00–2.99		4.00–6.99	1.00–1.99				
Above average	4.00–5.99	3.00–4.99		7.00–11.99	2.00–4.99				
High	6.00–12.99	5.00–7.17	2.00–5.99	12.00–22.99	5.00–11.99	1.15–1.56	1.14–1.56	1.14–1.56	1.29–1.70
Very high	13.00–40.00	7.20–12.00	6.00–28.00	23.00–80.00	12.00–21.00	1.57–5.00	1.57–5.00	1.57–5.00	1.71–5.00
Women									
Very low	0.00–0.99	0.00–0.99	0.00–0.99	0.00–0.99	0.00–1.99	1.00–1.13	1.00–1.28	1.00–1.13	1.00–1.17
Low									
Below average				1.00–4.99					
Average				5.00–7.99	2.00–3.99				1.18–1.52
Above average	1.00–1.99	1.00–1.99		8.00–14.99	4.00–6.99	1.14–1.28		1.14–1.42	1.53–2.28
High	2.00–4.99	2.00–3.99		15.00–30.99	7.00–13.99	1.29–1.92	1.29–1.99	1.43–2.13	2.29–3.46
Very high	5.00–40.00	4.00–12.00	1.00–5.00	31.00–80.00	14.00–21.00	1.93–51.00	2.00–5.00	2.14–5.00	3.47–5.00

AUDIT: TOT = total score; USU = alcohol abuse; PRO = problems due to abuse; CESD = total score on CESD; GAD-7 = total score on GAD-7; ACQ: TOT = total score, PHY = fear of physical symptoms, LC = fear of losing control.

In sum, the study aim is two-fold: we provide normative data for the AUDIT, CES-D, GAD-7, ACQ, and BSQ for the Surinamese population. Thus, we generated age- and gender-specific normative data for these five measures. Secondly, based on Item Response Theory models for their scoring, we made crosswalks (tables and figures) to convert scores on these measures into a common metric (normalized T-scores) and we established formulas to convert raw scores into T-scores. Thus, we aim to facilitate the interpretation of test results in research and clinical practice.

## Methods

### Participants

The study was conducted in two districts of Suriname, Paramaribo (the capital of Suriname), a predominantly urban district, and Nickerie, a predominantly rural district. Respondents were recruited by the census bureau of Suriname. A stratified sampling method was used by random selection of respondents from 12 resorts of Paramaribo and 5 resorts of Nickerie, assuring a balanced geographical distribution of respondents. There were 2,863 participants in the study (15), 1837 respondents with an urban background (Paramaribo 1,065 women and 772 men) and 1,026 participants with a rural background (Nickerie, 593 female and 433 male). All questionnaire data were collected by trained interviewers. We refer for more details on the study to Jadnanansing et al. (15). Table 2 provides demographic data for the participants.

**Table 2:** Demographic data for all respondents and for the urban and rural samples.

		Total		Paramaribo		Nickerie		χ^2^(df 1)	*p*
		N	%	N	%	N	%		
Gender	Female	1,658	57.9	1,065	58.0%	593	57.8%	0.002	0.92
	Male	1,205	42.1	772	42.0%	433	42.2%		
								χ^2^(df 5)	*p*
Age group	16–19	236	8.2	150	8.2%	86	8.4%	0.06	0.04
	20–29	590	20.6	401	21.8%	189	18.4%		
	30–39	567	19.8	370	20.1%	197	19.2%		
	40–49	609	21.3	361	19.7%	248	24.2%		
	50–59	577	20.2	364	19.8%	213	20.8%		
	60–69	284	9.9	191	10.4%	93	9.1%		
								χ^2^(df 2)	*p*
Education	Primary	1,076	42.0	569	37.0%	507	49.5%	0.21	0.001
	Secondary	1,060	41.3	764	49.6%	296	28.9%		
	Higher	428	16.7	206	13.4%	222	21.7%		

### Instruments

For all measurement instruments Dutch language versions were used.

#### Alcohol abuse and dependence: AUDIT

The Alcohol Use Disorders Identification Test AUDIT (16) was developed by the World Health Organization to screen for problematic alcohol use. The AUDIT is a 10-item screening test to assess alcohol consumption, drinking behavior and drinking related problems. Items are scored on a 5-point Likert frequency scale 0 “Not at all” to 4 “Daily or almost daily.” The total score (AUDIT_TOT) has a theoretical range of 0 to 40. The cut-off score used for increased risk for problematic alcohol consumption is 8 (17). Based on a considerable number of studies, Peng and colleagues concluded that the AUDIT comprises two factors, alcohol consumption (AUDIT_USE, items 1–3) and symptoms of alcohol dependence and problem consequences from drinking (AUDIT_PRO, items 4–10).

#### Depression: the CES-D

The Center for Epidemiological Studies-Depression (CES-D) scale was designed to measure the level of depressive symptomatology in the general population (18). Twenty items inquire about the frequency symptoms that occurred in the past week with response options from 0 “Not at all” to 3 “Nearly every day.” The total score ranges between 0 to 60 and the cut-off point that has been typically recommended for depression “caseness” is 16. However, more recently 20 has also been recommended as cut-off value (19).

#### Anxiety: GAD-7 and ACQ/BSQ

Two aspects common to anxiety were measured: generalized anxiety or excessive worry and fear of fear. Generalized anxiety and worry was measured with the GAD-7 (20). This is a widely used measure, recommended by the International Consortium of Health Outcome Measurements (ICHOM) for treatment outcome measurement in anxiety disorders Furthermore, it is routinely administered in all “Improving Access to Psychological Therapies” (IAPT) services in the UK (21). The GAD-7 comprises seven items describing feelings, such as “Trouble relaxing,” “Feeling nervous, anxious or on edge” and “Feeling afraid as if something awful might happen.” Items are scored on a 4-point Likert frequency scale (0 “Not at all” to 3 “Nearly every day”), resulting in a theoretical range in scores of 0 to 21. Kroenke et al. (22) suggested as cut-off scores for mild, moderate, and severe anxiety symptoms: 5, 10, and 15. When the GAD-7 is applied for screening, further evaluation is recommended when the score is 10 or higher (20).

Fear of fear was measured with the Agoraphobic Cognitions Questionnaire [ACQ; (23)] and the Body Sensations Questionnaire [BSQ; (23)]. The ACQ was devised to measure maladaptive thoughts about the possible consequences of panic (the cognitive aspect). On 14 items respondents rate the frequency of these thoughts when feeling anxious or frightened. Each item is rated on a 5-point Likert frequency scale, ranging from 1 “Thought never occurs” to 5 “Thought always occurs.” Next to a total score (ACQ_TOT), the ACQ measures two factors: ACQ_SC for social/behavioral concerns (e.g., loss of control, acting foolishly) and ACQ_PHY for physical concerns (e.g., having a heart attack, fainting). The scale discriminates well between patients and normal controls: Chambless et al. (23) reported a mean score of *M* = 2.32 (*SD* = 0.66) for outpatients with agoraphobia, and *M* = 1.60 (*SD* = 0.46) for a community sample. The BSQ measures fear of the bodily sensations which are commonly experienced during anxiety and panic attacks. The BSQ comprises 17 items, each describing a physical symptom, such as dizziness, palpitations, or breathlessness. Items are rated on a five-point scale for how much anxiety they provoke ranging from 1 “Not at all” to 5 “Extremely.” Chambless (23) reported a mean score of *M* = 3.05 (*SD* = 0.86) for outpatients with agoraphobia, and *M* = 1.80 (*SD* = 0.59) for a community sample. The Dutch version of the ACQ and BSQ have been psychometrically evaluated by Arrindell (24) and appeared reliable (internal consistency Cronbach’s α > 0.82 and α > 0.89 for the ACQ and BSQ, respectively) and had good test–retest reliability (Pearson PMC r > 0.79 for both scales.

### Statistical analysis

We calculated descriptive statistics, such as the mean, standard deviation, skewness, and kurtosis of all scale scores for the sample and compared scores by gender, age group, and urban or rural background with independent samples *t*-tests and Mann Whitney U tests. We also compared mean scores of the Suriname respondents to community samples of other countries/cultures. We established norm tables in order to give meaning to scale scores. Finally, we established for all scales cross-walk tables to convert raw scores to a common metric: T-scores (25). These T-scores were based on theta’s from IRT models. All analyses were performed with R. We used the mirt package of R, version 1.33.2 (26) to determine relevant item characteristics and to calculate scale scores (factor score) from item responses. We used the “Graded Response Model for polytomous items” with the Expected-A-Posteriori sum score (EAPsum) as estimator, in accordance with the approach chosen by the PROMIS group and proposed by Fischer and Rose (27). We used the mirt package to assess the fit of a unidimensional model for each (sub)scale that was analyzed.

We evaluated uniform and nonuniform DIF (28) for gender, age (recoded into a binary variable <45 and ≥45, and urbanicity (urban vs. rural). Both types of DIF were assessed with ordinal logistic regression (OLR) methods (29) using the R package lordif Version 0.3-3 (30). As measure of effect size, we used the change in McFadden’s pseudo *R^2^*, lavaan (version 06.5; (31)). We used the (scaled) fit statistics and set as requirements for unidimensionality the following the suggestion of 0.02 as critical value for rejecting the hypothesis of no DIF (30). We examined for each scale the fit of the graded response model with inspection of item parameters estimates using the item fit signed chi-square (S-χ^2^) statistic (32) as indicator of item misfit. Items with a S-χ^2^ *p* < 0.001 are considered to have a poor fit in the IRT model. The assumption of *monotonicity* was evaluated by examining graphs of item mean scores as a function of rest scores (total raw score minus the item score) using the R package Mokken (Version 23.0.6; (33). In addition, we evaluated the accompanying scalability coefficients (Mokken’s *H*) for the full scale and the individual items. Mokken’s *H* was interpreted as follows: 0.30 ≤ *H* < 0.40 low quality, 0.40 ≤ *H* < 0.50 moderate quality, and *H* ≥ 0.50 high quality (Mokken, 1971). Also, we investigated *local independence* (LID). Item pairs are locally independent when items show no association after controlling for the trait level. Investigation of LID was done with Yen’s *Q3* statistic (34) in mirt.

Finally, we created a cross-walk table and cross-walk figure to convert raw scores to IRT-based T-sores. We also established equations for this conversion with regression analysis (curve fitting). Linear, polynomial, exponential, logarithmic, power, rational, sigmoid, and hyperbolic equations (and exponential, logarithmic, and power equations with an added linear term) were fitted with Nonlinear Least Squares (nls and nls2) of the R package nlstools (version 2.0-0) (35). We compared the fit of these various equation by their Bayesian Information Criterion (BIC) value for each scale. The procedure is described in more detail and cross-validated by de Beurs et al. (36).

## Results

We first checked whether the samples of the included respondents were comparable to the populations of the two areas concerning gender and age. The gender distribution in the general population of Paramaribo (*N* = 140,679) is 51% women and 49% man; 53% is younger than 40 years old and 47% is 40 years or older. In the present sample a significantly different gender distribution was obtained: 58% women (χ^2^ = 35.6; df = 1; *p* < 0.001) and 54% of the respondents is ≥40 years (χ^2^ = 34.9; df = 1; *p* < 0.001). In Nickerie (*N* = 34,233) the gender distribution is 47% women and 53% men; 52% is younger than 40 years and 48% is 40 years or older. The gender distribution in the present sample was: 59% women (χ^2^ = 46.9; df = 1; *p* < 0.001) and 54% of the respondents ≥40 years (χ^2^ = 13.3; df = 1; *p* < 0.001). Thus, elderly women were somewhat overrepresented in our samples.

Table 2 presents an overview of demographic characteristics of the participants. There were differences between the urban sample and the rural sample in marital status, number of children ethnic background and work status, in age, educational level difference, but among the subsamples there were no differences in representation of the genders, with in both subsamples an equal overrepresentation of elderly women.

Next, we compared the scores obtained in the current Surinamese sample with other normative sample from the USA, Germany, and the Netherlands. Peng et al. (17) offer means for the AUDIT based on an analysis of AUDIT data from 15 countries. Lowe et al. (37) provided normative data for the GAD-7 from a large sample of the German population. In addition, we use data for the GAD-7 from a USA African-American sample reported in the study of Parkerson et al. (38). Bouwman et al. (39) collected CES-D data in a substantial Dutch population-based sample. Chambless obtained data on the ACQ and BSQ from a small sample of females (n = 21); Craske et al. (40) collected ACQ data from a student sample N = 173); de Beurs (41) obtained data from a representative sample of the Dutch general population n = 438, of which 263 were females, 60.0%).

Table 3 present mean scores (and SD’s) on the instruments from Surinamese respondents and from normative samples from the USA, the Netherlands and Germany. We compared mean scores in Table 3 by inspection only, as most means will differ statistically, given the large sample sizes. Scores of Surinamese respondents are lower regarding the total score for Alcohol abuse and dependence, use, and problems with alcohol (AUDIT) compared to the rest of the world according to the data of Peng et al. (17), especially among women. Depression scores are lower compared to the USA, but higher than in the Netherlands. Scores on the GAD are somewhat elevated compared to the German normative sample and comparable to the US. Scores on the ACQ and BSQ are similar to a Dutch normative sample, but lower compared to respondents from the USA. The anxiety scores on the GAD-7 are substantially elevated compared to the German population. Fear of fear according to the ACQ and fear of body sensations according to the BSQ is similar to the Netherlands, but lower compared to USA samples. Finally, the data reveal a substantial difference between men and women in problematic alcohol consumptions (males > females, as well as in depression and anxiety (females > males). The data also suggest a larger gender difference in Suriname compared to USA and European samples.

**Table 3:** Means and standard deviations on the instruments from Surinamese respondents and from other normative samples.

Scale	Region	N	All respondents		Males		Females	
			M	SD	M	SD	M	SD
AUDIT-TOT	Suriname	2,863	2.33	3.53	3.85	4.44	1.23	2.09
	World^1^	27,487	NA	NA	5.09	3.51	3.30	2.58
AUDIT-USE	Suriname	2,863	1.73	2.11	2.73	2.53	1.00	1.35
	World^1^		NA	NA	3.44	1.36	2.46	1.27
AUDIT-PRO	Suriname	2,863	0.61	1.90	1.12	2.54	0.23	1.10
	World^1^		NA	NA	1.58	2.79	0.72	1.82
CESD	Suriname	2,863	8.66	9.14	7.54	7.89	9.47	9.86
	USA^2^	747	10.60	11.20	NA		NA	
	Dutch^3^	2,667	7.38	7.55	6.25	6.76	8.41	8.05
GAD-7	Suriname	2,863	3.58	4.35	2.96	3.86	4.04	4.64
	German^4^	5,030	2.97	3.38	2.66	3.24	3.20	3.52
	USA^5^	103	3.76	3.96	NA		NA	
ACQ-TOT	Suriname	2,863	1.17	0.34	1.12	0.25	1.21	0.39
	USA^6^	173	1.55	0.42	NA		NA	
	Dutch^7^	438	1.23	0.34	1.21	0.34	1.24	0.35
ACQ-PHY	Suriname	2,863	1.15	0.36	1.09	0.26	1.19	0.42
	USA^7^	21	1.31	0.33	NA		NA	
	Dutch^8^	438	1.25	0.34	1.23	0.34	1.26	0.34
ACQ-LC	Suriname	2,863	1.19	0.41	1.15	0.34	1.23	0.45
	USA^7^	21	1.89	0.70	NA		NA	
	Dutch^8^	438	1.21	0.35	1.20	0.34	1.22	0.36
BSQ	Suriname	2,863	1.57	0.78	1.43	0.67	1.67	0.84
	USA^6^	173	1.80	0.59	NA		NA	
	Dutch^8^	438	1.47	0.54	1.52	0.51	1.51	0.52

AUDIT-TOT = total score; AUDIT-USU = alcohol abuse; AUDIT-PRO = problems due to abuse; CESD = total score on CESD; GAD-7 = total score on GAD-7; ACQ-TOT = total score on ACQ, ACQ-PHY = fear of physical symptoms, ACQ-LC = fear of losing control.

1 Peng et al. (17).

2 Choi et al. (12).

3 Bouwman et al. (39).

4 Löwe et al. (37).

5 Parkerson et al. (38).

6 Craske et al. (40).

7 Chambless et al. (23).

8 de Beurs (41).

Table 4 presents mean scores, SD’s, skewness and kurtosis of scale scores from the current sample. Most instruments yielded skewed and peaked frequency distributions of scores, due to an excess of low scores, especially on the AUDIT and ACQ (zero score-inflation).

**Table 4:** Means and standard deviations, range, kurtosis, and skewness of scores on the scales of Surinamese respondents.

Scale:	M	SD	Range	Skewness	Kurtosis
AUDIT-TOT	2.33	3.53	0–36	3.02	13.53
AUDIT-USE	1.73	2.11	0–12	1.80	3.73
AUDIT-PRO	0.61	1.90	0–24	4.82	31.61
CESD	8.56	9.06	0–55	1.71	3.22
GAD	3.85	4,35	0–21	1.71	2.85
ACQ-TOT	1.17	0.34	1–5	3.79	20.45
ACQ-PHY	1.15	0.36	1–5	4.09	22.16
ACQ-SC	1.19	0.40	1–5	3.46	15.34
BSQ	1.56	0.77	1–5	1.66	2.24

AUDIT-TOT = total score; AUDIT-USU = alcohol abuse; AUDIT-PRO = problems due to abuse; CESD = total score on CESD; GAD-7 = total score on GAD-7; ACQ-TOT = total score on ACQ, ACQ-PHY = fear of physical symptoms, ACQ-SC = social concerns.

Table 5 presents means by gender, age group and urban or rural background. We tested for differences with *t*-test and Mann–Whitney U tests, given the non-normal distribution of scores on some measures. There was a significant difference between men and women on all measures. Males scored higher on problematic alcohol use [*t*(2851) = 23.69, *p* < 0.001], but lower on depression and anxiety. Age groups also differed significantly on all measures, except on the CES-D [*t*(2851) = 0.36, *p* = 0.72] and the ACQ-TOT [*t*(2851) = 1.49, *p* = 0.14], with younger respondents having higher scores, across the board. Finally, the scores of the urban and rural background levels did not differ on most measures, except for small differences on the CES-D, ACQ physical concerns and BSQ with higher scores in the rural resorts. Most differences between means of subgroups were small, with the exception of the large gender difference on the AUDIT, and small to medium gender differences on the GAD-7, the ACQ, and the BSQ. These gender differences and differences between younger and older respondents justify the distinction of various groups for norms. Thus, we decided to provide separate norming tables for both genders and, in addition, we calculated T-scores separately for six age groups 16–19, 20–29, 30–39, 40–49, 50–59, 60 years and older.

**Table 5:** Means and standard deviations for men and women, younger and older, and urban and rural respondents, results of *t*-test and Mann Whitney U tests and effect size (Cohen’s d).

Scale	Men		Women		*t*(2851)	U	Cohen’s d
	M	SD	M	SD			
AUDIT-TOT	3.85	4.44	1.23	2.09	21.00***	554200***	0.84
AUDIT-USE	2.73	2.52	0.99	1.35	23.69***	553884***	0.97
AUDIT-PRO	1.12	2.54	0.23	1.10	12.61***	780209***	0.49
CESD	7.51	7.84	9.45	9.85	5.67***	1087175***	0.35
GAD	2,95	3.84	4.04	4.63	6.66***	1135590***	0.40
ACQ-TOT	1.12	0.25	1.21	0.39	6.96***	1152782***	0.50
ACQ-PHY	1.09	0.26	1.19	0.42	7.34***	1151628***	0.55
ACQ-SC	1.15	0.34	1.22	0.45	5.19***	1094452***	0.33
BSQ	1.43	0.67	1.66	0.83	8.03***	1178336***	0.49
	Age < 45		Age ≥ 45				
AUDIT-TOT	2.53	3.58	2.05	3.44	3.65***	554200***	0.14
AUDIT-USE	1.84	2.15	1.56	2.05	3.52***	553884***	0.13
AUDIT-PRO	0.69	1.93	0.48	1.85	2.91**	780209***	0.11
CESD	8.59	8.91	8.69	9.39	0.27	1087175***	0.01
GAD	3.74	4.29	3.35	4.43	2.36*	1135590***	0.09
ACQ-TOT	1.18	0.34	1.16	0.34	1.24	1152782***	0.05
ACQ-PHY	1.14	0.33	1.17	0.40	2.63**	1151628***	0.10
ACQ-SC	1.22	0.42	1.15	0.37	4.48***	1094452***	0.17
BSQ	1.62	0.79	1.49	0.75	4.04***	1178336***	0.15
	Urban		Rural				
AUDIT_TOT	2.37	3.47	2.26	3.47	0.78	955,729	0.03
AUDIT_USE	1.74	2.12	1.70	2.12	0.45	951,837	0.02
AUDIT_PRO	0.63	1.78	0.56	1.78	0.96	917,901	0.04
CESD	8.38	9.21	9.10	9.21	2.02*	878319*	0.08
GAD	3.59	4.42	3.56	4.42	0.16	956,285	0.01
ACQ_TOT	1.16	0.37	1.19	0.37	2.00	906,121	0.07
ACQ_PHY	1.14	0.40	1.18	0.40	2.81	888240**	0.10
ACQ_SC	1.19	0.42	1.20	0.42	0.86	922,268	0.03
BSQ	1.54	0.78	1.60	0.78	1.90	870388**	0.07

AUDIT-TOT = total score; AUDIT-USE = alcohol abuse; AUDIT-PRO = problems due to abuse; CESD = total score on CESD; GAD-7 = total score on GAD-7; ACQ-TOT = total score on ACQ, ACQ-PHY = fear of physical symptoms, ACQ-SC = social concerns.

* *p* < 0.05;

** *p* < 0.01;

*** *p* < 0.001

Table 1 offers meaning to scores on the instruments by providing cut-off scores for seven norm levels: very low (the lowest 5%), low (the next 15%), below average (20%), average (20%), above average (20%), high (15%), very high (5%). Differentiation among low scores is hard on several instruments. This is especially the case with the AUDIT-problem score, as it allows only a distinction between a very high score and every level below it.

Next, we established T-scores based on theta’s from IRT models for the instruments. First, we investigated the fit of IRT models to obtain factor scores. We inspected for each scale the Limited information goodness of fit test statistic that mirt provides. Results of these analyses are summarized in Table 6 (M2 and additional fit indices). The signed chi-square (S-χ^2^) statistic was calculated as indicator of item misfit. Some items were found with a statistically significant S-χ^2^ indicating poor item fit. Inspection of plots for item performance yielded satisfactory results. Plots for test information and empirical test plots (to check for unidimensionality) were inspected as well. Test were most informative in the theta = −0.5 to 2.5 range, which is due to high frequency of low scores in the present sample. The assumption of *monotonicity* was evaluated by examining graphs and we evaluated the accompanying scalability coefficients (Mokken’s *H*) for the full scale and the individual items. Most scales appeared to have low to moderate quality according to Mokken’s *H*. These results are presented in Table 6 as well.

**Table 6:** Information on IRT model fit indices and item fit statistics for the measurement instruments.

	χ^2^	*df*	*p*	RMSEA	SRMR	TLI	CFI	S_χ^2^ (item)	Mokken’s H	Highest Yen’s Q3
AUDIT	4.56	7	0.714	0.001	0.105	1.000	1.000	None	0.45	3 and 8 (0.26)
CESD	1230.63	130	<0.001	0.059	0.057	0.947	0.954	4, 8, 12, 16	0.27	12 and 16 (0.30)
GAD	NA	NA	NA	NA	NA	NA	NA	None	0.51	2 and 5 (0.26)
ACQ	476.48	35	<0.001	0.072	0.082	0.953	0.966	9	0,35	8 and 9 (0.30)
BSQ	762.52	68	<0.001	0.065	0.086	0.966	0.973	None	0.52	3 and 5 (0.53)

For subscales of measures (AUDIT_USE and AUDIT_PRO, ACQ_PHY and ACQ_SC) and for the GAD this information is not available due to insufficient degrees of freedom for the analyses to run. RMSEA = root mean square error of approximation; SRMR = standardized root mean square residual; TLC = Tucker-Lewis Index; S_ χ^2^ (item) = items where misfit according to the item fit indicator signed chi-square statistic is established; Mokken’s *H* = scalability coefficients; Yen’s Q3 = item pairs with highest local dependence.

Furthermore, uniform and nonuniform DIF was investigated for gender, age, urbanicity. Significant DIF was only found for the ACQ, where two items were flagged (items 4 and 9). Local independence (LID) of item pairs was investigated with Yen’s Q3 that mirt provides and this information is included in Table 6. As suggested by Smits et al. (42), model fit was evaluated with Cohen’s (43) rules of thumb to interpret effect size; Q3 values between 0.24 and 0.36 imply moderate deviations, Q3 values above 0.37 imply large deviations. For each scale only a few item pairs with a high Q3 value were found. Table 6 also shows the item pairs with the highest Q3 value for each instrument.

Finally, we established equations to calculate normalized T-scores, which are included in a note under Table 7. For most scales, cubic polynomial functions fitted best. We validated these formulas by investigating the correspondence between theta-based T-scores and calculated T-scores with intraclass correlation coefficients (all in the range of ICC = 0.97 to 0.99) and inspected Bland–Altman plots. Formulas to calculate T-scores for the genders and age groups were also established and can be obtained from EdeB. Finally, we established T-scores based on theta’s from IRT models for the instruments. First, unidimensionality of the factor structure of each subscale was investigated by comparing fit indices with the preset requirements (CFI > 0.95, RMSEA < 0.08, and SRMR < 0.06). Most scales showed adequate fit to a unidimensional model (see Table 6). Scales with fit indices that did not meet the criteria were: CES-D (SRMR = 0.071), ACQ_TOT (SRMR = 0.085), and the BSQ (CFI = 0.81, RMSEA = 0.085). For the CES-D this may be due to the four positively stated items in this questionnaire, as these were the items showing misfit according to item fit indicator signed chi-square (S-χ^2^) statistic (32).

**Table 7:** Crosswalk table from raw scores to theta-based T-scores.

AUDIT						CESD		GAD-7		ACQ				BSQ	
RS	TOT	RS	USE	RS	PRO	RS	TOT	RS	TOT	RS	TOT	PHY	SC	RS	TOT
0	40.6	0	40.8	0	47.5	0	37.0	0	39.7	1.00	43.5	45.6	44.9	1.00	39.5
1	48.6	1	48.8	1	59.9	2	44.5	1	46.1	1.07	50.8			1.06	45.5
2	53.8	2	54.5	2	60.6	4	47.2	2	49.6	1.14	54.4	54.5	53.7	1.12	48.1
3	56.8	3	57.8	3	65.0	6	49.1	3	51.2	1.21	56.3			1.18	49.6
4	58.8	4	59.9	4	60.4	8	52.4	4	54.1	1.29	57.6	58.8	57.4	1.24	50.6
5	60.1	5	62.5	5	65.9	10	53.9	5	55.4	1.33		61.4	57.3	1.25	51.6
6	61.4	6	64.4	6	67.4	12	53.9	6	57.2	1.43	60.2	59.8	58.9	1.35	52.7
7	62.9	7	66.7	7	68.5	14	57.1	7	58.5	1.50	61.8	58.2	55.8	1.41	53.5
8	63.4	8	68.0	8	69.6	16	58.2	8	59.4	1.57	62.3	61.5	60.0	1.53	54.7
9	64.2	9	70.5	9	71.4	18	60.0	9	60.6	1.71	63.6	64.6	63.2	1.76	56.5
10	65.8	10	72.1	10	71.3	20	61.8	10	61.8	1.86	64.1	64.2	64.1	1.94	57.9
11	65.9	11	73.9	11	70.5	22	61.9	11	63.2	2.00	66.6	66.4	64.5	2.00	58.7
12	66.6	12	78.9	12	73.1	24	63.5	12	64.2	2.14	66.8	66.0	65.3	2.18	59.1
13	66.6			13	74.2	26	65.0	13	65.3	2.29	67.8	68.4	66.4	2.40	60.9
15	68.8			14		28	65.9	14	66.5	2.43	68.2	69.2	67.8	2.71	61.9
16	68.5			15		30	66.5	15	68.1	2.50	70.6			2.82	62.6
18	71.0			16	76.0	32	67.7	16	69.4	2.57	70.5	70.3	68.8	3.00	63.5
19	72.0			17		34	69.1	17	70.2	2.64	72.0			3.06	63.9
20	71.8			18	79.8	36	70.4	18	72.4	2.71	72.2	71.3	69.0	3.18	64.3
23	73.9			19	80.0	38	72.4	19	73.7	2.79	72.9			3.24	64.9
25	74.8			20	80.1	40	73.0	20	74.4	2.86	73.3	72.0	71.2	3.47	65.8
29	78.4			21		42	72.6	21	78.0	3.00	72.8		71.7	3.53	66.4
30	78.8			22		44	76.9			3.14	74.9	73.7	72.5	3.76	67.1
36	85.7			23		46	73.9			3.29	76.4	74.0	73.9	3.82	68.0
40				24	86.5	48	79.3			3.64	79.6			4.00	68.1
						50	79.6			3.86		78.6	80.6	4.18	71.1
						52	80.2			3.93	79.2			4.35	72.7
						54				4.00	79.3	77.8	76.8	4.53	75.6
						55	83.0			4.43		81.4		4.82	77.7
						56				5.00	94.2	89.6	88.5	5.00	82.0

RS = raw score; AUDIT-TOT = total score; AUDIT-USU = alcohol abuse; AUDIT-PRO = problems due to abuse; CESD = total score on CESD (only even raw scores are shown); GAD-7 = total score on GAD-7; ACQ-TOT = total score on ACQ, ACQ-PHY = fear of physical symptoms, ACQ-SC = social concerns. NB. Formulas to calculate T-scores for respondents, irrespective of gender: AUDIT-TOT: 0.0022x^3^–0.134x^2^ + 3.06x + 46.5; AUDIT-USE: 0.0403x^3^–0.858x^2^ + 7.59x + 41.5; AUDIT-PRO: 0.0058x^3^–0.228x^2^ + 3.57x + 53.6; CESD: 0.00036x^3^–0.0343x^2^ + 1.592x + 40.4; GAD: 0.0049x^3^–0.175x + 3.19x + 42.4; ACQ-TOT: 1.3931x^3^–12.973x^2^ + 45.74x + 15.8; ACQ-PHY: 1.2714x^3^–12.207x^2^ + 43.65x + 17.7; ACQ-SC: 1.0683x^3^, −10.426x^2^ + 39.28x + 19.3; BSQ: 1.2126x^3^–10.931x^2^ + 36.86x + 18.6.

**Figure 1:**
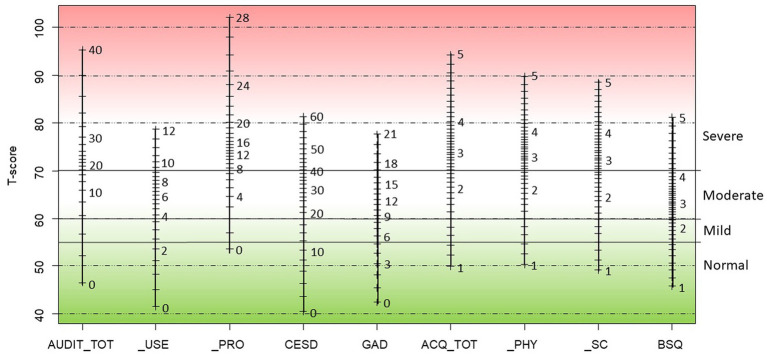
Crosswalk from raw scale scores to T-scores for the AUDIT, CES-D, GAD-7, ACQ, and BSQ.

Figure 1 shows for all scales the correspondence between raw scores on the scales and T-scores. Table 7 can be used to convert raw scores of all measures and subscales into T-scores. Formulas to calculate these T-scores are included in the note under Table 7. Formulas to calculate T-scores for the genders and age groups can be obtained from EdeB.

Figure 1 displays raw scores based on summed item scores on the scales and how they relate to the T-score metric. As can be seen in Figure 1, the original raw scores show a difference in interval width, which illustrates the non-normal distribution of these scores. After conversion to T-scores the score intervals become equally spaced on the Y-axis. Figure 1 can also be used to convert raw scores of all measures and subscales into T-scores. It is based on calculated T-scores, applying the formulas from the note under Table 7.

## Discussion

Data from a large representative sample from the general population of Suriname were collected to compare scores with other populations (from the USA and Europe) and to obtain norms on commonly used measures for alcohol use, depression and anxiety. Generally, scores appeared comparable to what has been found with these instruments on other continents. Also, we found differences between men and women and between younger and older respondents, similar to what has been reported in the literature (44, 45). Men report higher and more problematic alcohol use, which is cross-culturally a consistent finding (46). However, at least in the USA, the gender gap is closing as the difference is smaller for later birth cohorts (47). In an exploratory analysis, we investigated the effect of gender and age conjointly and we did find a significant interaction effect (alcohol use diminishes with age faster for men compared to women), but the effect size of this interaction was rather small (*η^2^* = 0.007).

Surinamese women reported higher levels of depression and anxiety compared to men. Regarding depression, Stevenson and Wolfers (48) mentioned in a review on gender studies into well-being the apparent paradox that for women living conditions have improved over the last 30 years, but subjective well-being has declined, both in absolute numbers and relative to men. In line with their findings, we also found an effect of age, with the oldest age group scoring lower on the CES-D. Moreover, according to our findings, the gender gap was larger in Paramaribo than in Nickerie, because rural men tended to have elevated scores on the CES-D, bringing their score closer to the score of women. Regarding anxiety, men had lower scores and may indeed experience less anxiety, but this gender difference may have been amplified by a reporting bias: stereotypes and socialization make that men are less inclined to acknowledge experiencing fear or anxiety (44), especially when data are gathered by (female) interviewers. Further research is needed to explain these gender and age differences. However, the small to medium sized differences between men and women regarding depression and anxiety justify the use of distinct cut-off values for caseness, distinct norms, and distinct cross walk tables to T-scores. However, the user of test results should be aware that T-scores calibrated on such subgroups will no longer reveal any difference between these subgroups.

Based on expert judgement, the PROMIS group has provided guidelines for the interpretation of scores on the T-score metric and proposed the following cut-points on the T-score metric: <55 normal; 55–60 mild; 60–70 moderate; >70 severe (49). Scores of 55 and higher are reason for concern and above 60 a moderate severity level of depression or anxiety is reached. Application of these values coincides well with known cut-off values on the measures investigated in this study. The raw-scores and T-scores that were used to make Figure 1 correspond well with what is published in the research literature. If we inspect the scores on the depression scale, the cut-off of 16 for “caseness” on the CES-D corresponds to a cut-off of *T* = 56.2 in the USA sample (12) and a score of *T* = 58.6 in our sample. A comparison of raw scores and T-scores at cut-off values for the GAD-7 anxiety scale yields similar results. Schalet et al. (13) provided a crosswalk table for the GAD-7 based on the US community sample used to calibrate PROMIS-instruments and their values coincide well with our present findings. If we look at the correspondence of recommended cut-off points for “caseness,” a score of 10 on the GAD-7 corresponds to a T-score of 62.3 in the USA sample and a T-score of 61.7 in our present sample. These values on the T-score scale (61.7 and 58.6 for the GAD-7 and CES-D, respectively) also underscore the appropriateness of cut-off scores for “caseness” on the T-score metric as suggested by the PROMIS group. De Beurs and colleagues proposed to use 55 as cut-off point for “caseness” in the Netherlands (50). However, a more formal evaluation of cut-off values awaits further investigation and requires information on the clinical status of respondents. The present study did not collect such data.

Finally, comparison of the norms in Table 1 and the results after conversion of raw scores to T-scores reveal the increased informative value of T-scores. The information in Table 1 results from binning raw scores into seven categories (very low for the lowest 5%, low for the next 15%, below average for the next 20%, average for the next 40 to 60%, etc.) Thus, Table 1 gives meaning to scores in a categorized manner, basically converting raw scores to percentile scores and binning these in seven categories. In contrast, T-scores are continuous. For instance, norms on the ACQ for men allow us to distinguish only three levels: very high, high, and everyone else with a lower than high score; T-scores yield much more detailed information. On the other hand, this may also give rise to a false precision level. For instance, scores on the AUDIT (with a theoretical range of 0 to 40) are highly skewed to the right with many respondents obtaining the lowest possible score of 0 (34%) and most respondents have a score of 6 or lower (90%). Thus, only few respondents have high scores and most score in a broad category of very low to average. According to Figure 1 these respondents obtain T-scores from 46.5 to 60.5. Figure 1 also reveals that the evaluated measures are predominantly useful for the pathological range; as noted before, especially the ACQ and the BSQ do not distinguish well in the healthy range as these instruments assess aspects of anxiety mainly found in patients with panic disorder and evoke a 0-score from most community-based respondents, a phenomenon also known as zero inflation. This also explains the high T-score value for the lowest possible scores for most instruments. T-scores usually range from 20 to 80, which includes 99.7% of the cases when scores are distributed normally. However, application of these clinical measures in the general population yields T-scores in the range of 40 to 85 or higher.

Strength of the present study: a substantial number of respondents from the Surinamese population were included in the study, stratified to include urban as well as rural respondents, allowing us to establish norm for both genders and various age groups. A traditional approach to norming instruments was combined with a more modern IRT based conversion of scores to T-scores. This worked out well for most instruments.

Limitations: Some instruments scores were highly skewed and peaked due to the large proportion of respondents with the lowest possible score on these measures. This is a common finding when self-report instruments for psychopathological constructs are administered in population samples. The requirement of a normal distribution of scores for some of the statistical tests we used, such as *t*-test comparing subgroups in the sample, was not met on most scores. However, the nonparametric alternative statistical test (Mann Whitney U test) resulted in highly similar findings. Furthermore, as mentioned in the results section, some scales (e.g., the CES-D, ACQ_TOT, and BSQ) did not meet all the requirements of good fit of IRT modelling. When this is the case, revision of the item content or revision of the scoring of items or scales may be in order. Revising internationally established instruments would be beyond the scope of the present study. Nevertheless, due to insufficient fit of IRT models, the resulting factor scores may be biased. Alternative approaches to obtain T-scores should be considered, such as percentile rank score conversion (51) or regression-based norming (52).

## Conclusion

In sum, the present findings illustrate that internationally used cut-of values on self-report measures for case finding (in score on the original metrics and on the T-score metric) are appropriate for the population of Suriname. For most instruments, cut-off values for caseness for raw scores correspond well to generally recommended cut-of values for T-scores. T-scores are a convenient way to express how extraordinary a raw test score is on a continuous scale with equal intervals and T-scores are recommended to be used as a common metric for test results. In future studies, additional screeners may be evaluated on their utility in the Suriname context, such as screeners for other substance use disorders, adult Attention Deficit and Hyperactivity Disorder, and PostTraumatic Stress Disorder, which may otherwise easily remain undetected. Furthermore, these measurement instruments should be employed for mental health triage and routine outcome monitoring. Finally, their application may stimulate dissemination and use of (guided) self-help eMental-Health applications on smart-phones. This may help to bridge the existing treatment gap in Suriname, where stigma around mental health problems is still widespread and resources for mental health care are scarce.

## Data availability statement

The raw data supporting the conclusions of this article will be made available by the authors, without undue reservation.

## Ethics statement

The studies involving human participants were reviewed and approved by Ministry of Public Health of Suriname (#VG2014-09). The patients/participants provided their written informed consent to participate in this study.

## Author contributions

EB processed the data and drafted the manuscript. RJ co-designed the study, supervised the data acquisition in Nickerie, and critically reviewed the manuscript. KE collected the data and critically reviewed the manuscript. RB reviewed the study design and critically reviewed the manuscript. MB critically reviewed the manuscript. JP processed the data and critically reviewed the manuscript. JD supervised the whole study and critically reviewed the manuscript. All authors contributed to the article and approved the submitted version.

## Funding

Funding for data collection for this study was obtained from the Dutch Ministry of Foreign Affairs under the project title “Dwarkasing R. and de Jonge M. (2014). Onderzoek naar alcoholgebruik, angst en depressieve klachten in Suriname, en aanbieden van zorg op maat en geïndiceerde e-mental health. Paramaribo, Amsterdam.”

## Conflict of interest

The authors declare that the research was conducted in the absence of any commercial or financial relationships that could be construed as a potential conflict of interest.

## Publisher’s note

All claims expressed in this article are solely those of the authors and do not necessarily represent those of their affiliated organizations, or those of the publisher, the editors and the reviewers. Any product that may be evaluated in this article, or claim that may be made by its manufacturer, is not guaranteed or endorsed by the publisher.

## Abbreviations

ACQ-PHY: Agoraphobic cognitions questionnaire – physical concerns
ACQ-SC: Agoraphobic cognitions questionnaire – social/behavioral concerns
ACQ-TOT: Agoraphobic cognitions questionnaire – total score
AUD: Alcohol use disorders
AUDIT_PRO: Alcohol use disorders identification test, problems
AUDIT_TOT: Alcohol use disorders identification test, total score
AUDIT_USE: Alcohol use disorders identification test, use score
BSQ: Body sensations questionnaire
CES-D: Center for epidemiological studies-depression
CFI: Comparative fit index
CMD: Common mental disorders
EAP: Expected A-posteriori
GAD-7: Generalized anxiety disorder
IAPT: Improve access to psychological therapies
ICHOM: International consortium of health outcome measurements
LMIC: Low-to-middle income countries
PROMIS: Patient reported outcome measurement information system
RMSEA: Root mean square error of approximation
SRMR: Standardized root mean square residual
